# Addressing Challenges in Cerebral Aneurysm Management: Strategies to Enhance Patient Outcomes

**DOI:** 10.3390/jcm13082308

**Published:** 2024-04-16

**Authors:** Ramazan Jabbarli

**Affiliations:** Department of Neurosurgery & Spine Surgery, University Hospital Essen, 45147 Essen, Germany; ramazan.jabbarli@uk-essen.de

We are pleased to present a Special Issue dedicated to addressing the current challenges in the management of cerebral aneurysms (CA). This Special Issue comprises ten publications covering various topics related to the diagnosis and treatment of CA. A detailed list of contributions is provided at the end of this article.

Cerebral aneurysms (CA) represent saccular or fusiform enlargements of intracranial arterial vasculature, believed to develop under the prolonged influence of multiple endogenous and exogenous factors, the entirety of which remains incompletely elucidated [1]. CA is present in approximately 3% of the adult healthy population as small vascular lesions with an average diameter of 6–7 mm [2,3]. CA typically remains asymptomatic throughout an individual’s lifetime. However, the rupture of a CA leads to an aneurysmal subarachnoid hemorrhage (SAH), a devastating form of hemorrhagic stroke associated with high morbidity and mortality rates [4]. A myriad of complications occurring at different stages of SAH significantly contribute to the poor outcomes observed in individuals with ruptured CA [5,6,7].

Among the various pathophysiologic pathways implicated in CA development, neuro-inflammation is recognized as a key process driving CA genesis [8]. To shed further light on the role of neuro-inflammation in the pathophysiology of CA and its complications, Wach et al. (Contribution 1) conducted a systematic review and meta-analysis focusing on anti-inflammatory drug therapy in SAH. Their findings suggest that anti-inflammatory therapy in SAH patients may improve neurological outcomes without increasing mortality rates. They recommend further investigation through prospective multicenter randomized studies to evaluate the effects of inflammation management in SAH.

Given the poor outcomes associated with SAH, the preventive treatment of rupture-prone CA before the occurrence of a bleeding event is crucial for achieving favorable treatment results [9,10]. However, not all CA ruptures, and there is a significant risk of peri-procedural complications during CA treatment [11]. Thus, the proper identification of rupture-prone CA and the selection of individuals who may benefit from preventive CA treatment pose significant challenges in CA management. To facilitate this selection process, several scores assessing rupture risk have been developed in recent years [12,13]. Two studies by Wójtowicz et al. (Contributions 2 and 3) focused on the challenges in managing unruptured CA, particularly those located at the anterior communicating artery. These studies evaluated long-term functional outcomes following conservative and invasive management approaches and validated the accuracy and clinical applicability of different risk scores in their cohort.

In cases requiring CA treatment, optimizing the treatment process to prevent iatrogenic complications and achieve sustainable treatment outcomes is of paramount importance [10,14]. Endovascular treatment modalities have shown consistent progress over the past three decades, demonstrating improved applicability for various CA locations and morphologies [15]. Shüngel et al. (Contribution 4) present a comparative study on the use of distal flow diversion with anti-thrombotically coated and bare metal low-profile flow diverters in this issue.

As previously mentioned, SAH poses substantial morbidity and mortality risks despite maximal treatment efforts. Early and delayed complications of SAH, along with its initial severity, are major determinants of patient outcomes [5,6,7]. This Special Issue focuses particularly on studies addressing different aspects of SAH management, which play crucial roles in patient outcomes. In particular, Alsbrook et al. (Contribution 5) provide a systematic review of the pathophysiological backgrounds, diagnostic approaches, and therapeutic targets for managing early and delayed brain injuries after SAH, emphasizing perspectives for future experimental and clinical research.

The significance of detailed analysis of initial computed tomography scans for predicting complications and outcomes after SAH ([7,16], Contribution 6) is demonstrated in a study by Said et al. (Contribution 6). They show that not only the extent of intracranial bleeding but also the morphology of the ventricular system may serve as valuable radiographic markers for early recognition of complications and poor outcomes in SAH.

The use of anticoagulants before or during SAH can influence treatment outcomes, necessitating a detailed analysis. Veldeman et al. (Contribution 7) compare the impact of direct oral anticoagulants and vitamin K antagonists on the initial clinical and radiographic severity of SAH, as well as patient outcomes.

Güresir et al. (Contribution 8) and Vatter et al. (Contribution 9) present two studies from their center comparing conservative and invasive management options for cerebral vasospasm, addressing different treatment approaches and their effects on the occurrence of delayed cerebral ischemia and poor functional outcomes after SAH.

Proper intensive care management is essential for preventing many complications and irreversible brain damage after CA rupture. Anemia is a common condition following SAH, particularly in severely affected patients during the first weeks after the bleeding event ([17], contribution 10). Despite the lack of specific guidelines for anemia treatment after SAH, its management remains a highly debated topic in intensive care units. To address this issue, Said et al. (Contribution 10) analyze the effect of anemia on the course and outcome of SAH and propose recommendations for anemia management in SAH patients.

In summary, the studies included in this Special Issue underscore the importance of early recognition of different complication patterns after SAH. The insights gained from these studies may aid in establishing individualized treatment approaches for at-risk patients, thereby improving neurological outcomes. Additionally, in cases involving unruptured CA, precise patient selection for preventive interventions, coupled with efforts to improve intra-procedural safety and ensure the long-term sustainability of treatment outcomes, is imperative. These measures are vital in light of increasing life expectancy. Further research is warranted to corroborate the findings reported in this Special Issue.
